# Increased long noncoding RNA SNHG20 predicts poor prognosis in colorectal cancer

**DOI:** 10.1186/s12885-016-2719-x

**Published:** 2016-08-19

**Authors:** Cong Li, Li Zhou, Jun He, Xue-Qing Fang, Shao-Wen Zhu, Mao-Ming Xiong

**Affiliations:** 1 Department of Minimally Invasive Surgery, The People’s Hospital of Chizhou, Chizhou, 247000 China; 2 Department of General Surgery, First Hospital Affiliated to Anhui Medical University, Hefei, 230022 China

**Keywords:** Long noncoding RNA, SNHG20, Colorectal cancer, Cell cycle

## Abstract

**Background:**

Long noncoding RNAs (lncRNAs) have been suggested to be involved in the development and progression of malignancies. However, the investigation of small nucleolar RNA host gene 20 (SNHG20) on cancer progression remains unknown. The present study aims to explore the clinical significance of SNHG20 and its potential molecular mechanism in colorectal cancer (CRC).

**Methods:**

Quantitative real-time PCR (qRT-PCR) was used to measure the SNHG20 expression in a total of 107 CRC tissues and CRC cell lines. Loss of function approach was employed to explore the biological roles of SNHG20 in vitro. Its potential molecular mechanism was further verified by western blotting and qRT-PCR.

**Results:**

The results suggested that SNHG20 expression was significantly upregulated in CRC tissues compared to corresponding normal tissues from 107 CRC patients. High expression of SNHG20 was remarkably associated with advanced TNM stage in patients with CRC. Multivariate analyses unraveled that SNHG20 expression was an independent prognostic factor for overall survival in CRC patients. Further functional assays revealed that knockdown of SNHG20 suppressed cell proliferation, invasion and migration, and cell cycle progression in CRC cells. Moreover, SNHG20 regulated cell growth through modulation of a series of cell cycle-associated genes.

**Conclusions:**

Our findings suggest that dysregulation of SNHG20 participates in CRC progression and may serve as a potential therapeutic target in CRC patients.

## Background

Colorectal cancer (CRC) is the second most common in females and the third most frequent cancers in males, with an incidence in Europe of 471000 new cases and 228000 deaths in 2012 worldwide [1]. CRC is becoming as one of the most common malignancies and the fifth major cause of cancer-associated deaths in China [2]. Mortality caused by CRC in developed countries is declining, but there is a rapidly rising trend in China [3]. Despite improvements achieved in surgical resection and adjuvant chemotherapies, the 5-year survival rate of CRC patients remains unsatisfied [4]. Moreover, the 5-year survival rate of patients with resectable colorectal liver metastases is > 40 % but < 10 % in those with unresectable colorectal liver metastases [5]. Local and systemic metastases are the major causes for unsatisfactory outcomes of CRC patients. Currently, no widely approval parameter is used to offer a reliable information for clinical outcomes of patients with CRC [6]. Therefore, identification of effective carcinogenesis-associated molecular biomarkers that significantly unravel the clinical characteristics and implications of CRC is an important purpose of CRC investigation.

It is well known that long noncoding RNA (lncRNA) is transcribed RNA molecules more than 200 nucleotides and lack protein-coding potential [7]. LncRNAs are frequently presented as a disease-, tissue-, or stage-specific manner [8]. Multiple lines of evidence have indicated that lncRNAs, functioning as oncogenes or tumor suppressors, play important roles in the modulation of cellular processes, such as differentiation, proliferation, and metastasis [9]. Several specific lncRNAs have been increasingly considered as diagnostic or prognostic cancer biomarkers, including in CRC [10–12]. For example, metastasis-associated lung adenocarcinoma transcript 1 (MALAT1), a well-known lncRNA, is markedly overexpressed in CRC and has been suggested as a diagnostic cancer indicator [13–15]. Another characterized lncRNA, Hox transcript antisense intergenic RNA (HOTAIR), is also overexpressed in colorectal cancer, combines with PRC2 (Polycomb Repressive Complex 2) and changes the regulation of genes, leading to aberrant histone H3K27 methylation and further facilitating cancer progression and metastasis [11, 16, 17]. However, for all we know, the involvement of lncRNAs in CRC disease and prognosis is just starting to be investigated.

Small nucleolar RNA host gene 20 (SNHG20, GenBank Accession ID NR_027058.1) localized at 17q25.2 is originally identified in hepatocellular carcinoma (HCC) and suggested to be overexpressed in 2 HCC cohorts and TGCA dataset [18]. Moreover, SNHG20 expression may serve as a useful prognostic factor for patients with HCC [18]. However, its potential prognostic value and biological function in CRC have not yet been explored. In our current study, we first identified that SNHG20 overexpression was associated with aggressive phenotypes of CRC and worse outcomes in CRC patients. Further function experiments in vitro suggested that suppression of SNHG20 blocked cell proliferation, migration, invasion and cell cycle progression. Moreover, knockdown of SNHG20 affected the expression of cell cycle-associated genes in CRC cells. Taken together, these data suggest that SNHG20 participates as a noncoding oncogene in CRC carcinogenesis and progression.

## Methods

### Patients and specimens

All aspects of this study were approved by the Ethics Board of First Hospital Affiliated to Anhui Medical University. The written informed consents were obtained from all enrolled patients, and all relevant investigations were performed according to the principles of the declaration of Helsinki.

A total of 107 CRC paired tissue specimens (tumor and non-tumor tissues) were collected and histologically confirmed by a pathologist at The People’s Hospital of Chizhou or First Hospital Affiliated to Anhui Medical University, from January 2006 to January 2011. Corresponding normal tissues were taken 5–10 cm away from the edge of the tumor and contained no obvious tumor cells by the pathologist. Tissue specimens were immediately kept in RNA keeper tissue stabilizer (Vazyme, Nanjing, China) after surgery and then stored in −80 °C until RNA extraction. None of patients received anti-cancer treatment before surgery. Other types of cancers were not observed before operation. The detailed information on clinical characteristics of CRC patients in the present study is shown in Table 1. We also performed a follow-up study including physical examination, laboratory analysis, and colonoscopy if necessary.

**Table 1:** Correlation between the clinicopathological factors and expression of SNHG20

Factors	Tumor low expression (*n =* 53) *N* (%)	Tumor high expression (*n =* 54) *N* (%)	*P* ^*a*^
Sex			
Male	37 (69.8)	38 (70.4)	0.949
Female	16 (30.2)	16 (29.6)	
Age (years)			
< 65	25 (47.2)	26 (48.1)	0.919
≥ 65	28 (52.8)	28 (51.9)	
Tumor location			
Colon	32 (60.4)	42 (77.8)	0.051
Rectum	21 (39.6)	12 (22.2)	
TNM			
I-II	30 (56.6)	20 (37.0)	**0.043**
III-IV	23 (43.4)	34 (63.0)	
T			
T1-T2	11 (20.8)	3 (5.6)	**0.020**
T3-T4	42 (79.2)	51 (94.4)	
N			
N0	30 (56.6)	30 (55.6)	0.913
N1-N2	23 (43.4)	24 (44.4)	
M			
M0	44 (83.0)	32 (59.3)	**0.007**
M1	9 (17.0)	22 (40.7)	
Grade^b^			
G1-G2	46 (86.8)	40 (74.1)	0.098
G3	7 (13.2)	14 (25.9)	
CEA			
< 5 ng/mL	19 (35.8)	8 (14.8)	**0.012**
≥ 5 ng/mL	34 (64.2)	46 (85.2)	

Statistical significance is highlighted by bold font

*T* depth of tumor, *N* lymph node, *M* distant metastasis, *CEA* carcino-embryonic antigen

^a^Two-sided chi-square test

^b^Grade 1 and 2 stand for high or middle differentiated tumor, grade 3 stands for poorly differentiated tumor

### Cell culture

Human normal intestinal epithelial cell line FHC and CRC cell lines HCT8, HT29, HCT116, SW480, LOVO were purchased from a cell bank at Chinese Academy of Sciences (Shanghai, China). All cell lines were cultured in RPMI 1640 medium (Gibco, MD, USA) contained 10 % fetal bovine serum (HyClone, Logan, USA) and 100 U/ml streptomycin/penicillin (Gibco, MD, USA). The cells were maintained in a humidified atmosphere containing 5 % CO_2_ at 37 °C.

### RNA isolation and quantitative real-time PCR

Total RNA was extracted from CRC tissues with TRIzol reagent (Invitrogen, Carlsbad, CA, USA) according to the manufacturer’s protocols. The cDNA was synthesized from 1 μg of total RNA in a final volume of 20 μl using a PrimeScript RT reagent Kit with gDNA Eraser (Takara, Dalian, China). Its synthesis was conducted at 37 °C for 15 min, then 85 °C for 5 s according to the experimental protocols. Quantitative real-time PCR (qRT-PCR) was performed using a SYBR Premix EX Taq™ Kit (Takara, Dalian, China) by an ABI 7500 Real-Time PCR system (Applied Biosystems, Foster City, USA). GAPDH was employed as an internal control. Primer sequences of SNHG20: F, 5′-ATGGCTATAAATAGATACACGC-3′ and R, 5′-GGTACAAACAGGGAGGGA-3′; p21: F, 5′-CAGAGGAGGCGCCATGT-3′, R, 5′-GGAAGGTAGAGCTTGGGCAG-3′; CCNA1: F, 5′-ATTCATTAAGTGAAATTGTGC-3′ and 5′-CTTCCATTCAGAAACTTATTG-3′. GAPDH: F, 5′-ACAGTCAGCCGCATCTTCT-3′ and R, 5′-GACAAGCTTCCCGTTCTCAG-3′. The reaction was conducted in a reaction volume of 20 μl as the following processes: initial denaturation at 95 °C for 30 s, followed by 40 cycles for 95 °C for 5 s, 60 °Cfor 30 s. Fold changes were calculated using a relative quantification (2^-∆∆Ct^).

### RNA interference

For knockdown of SNHG20 expression, small interfering RNAs that targeted SNHG20 (si-SNHG20-1, si-SNHG20-2) and a scrambled negative control (si-NC) were purchased from Shanghai GenePharma Co. (Shanghai, China). The sequences of siRNAs (si-SNHG20-1, 5′-GCCUAGGAUCAUCCAGGUUTT-3′; si-SNHG20-2, 5′-GCCACUCACAAGAGUGUAUTT-3′) and si-NC were chemically synthesized and transfected into LOVO/SW480. Briefly, a total of 1.0 × 10^5^ cells were seeded in 6-cm culture dishes overnight and subsequently transfected with siRNAs described above by the Lipofectamine 2000 (Invitrogen, Carlsbad, CA) for 48 h. Transfected cells were then subjected into further functional assays and RNA/protein extraction.

### Cell proliferation assay

2-(2-Methoxy-4-nitrophenyl)-3-(4-nitrophenyl)-5-(2,4-disulfothenyl)-2H-tetrazolium salt (CCK-8, Dojindo, Rockville, USA) assay was performed to assess cell viability according to the manufacturer’s instruction. Briefly, transfected cells were seeded in 96-well plates (1.0 × 10^3^/per well). CCK-8 solution was added to each well, and cells were maintained for 1 h. The absorbance of each well was measured at 450 nm by a microplate reader victor (Enspire 2300 Maltilabel Reader, PerkinElmer, Singapore).

### Cell apoptosis assay

Cell apoptosis was analyzed using flow cytometry after staining with propidium iodide (PI) and Annexin V-FITC (BD Bioscience, CA, USA). Cells were transfected with si-NC or si-SNHG20-1 in 6-well plate. Cell apoptosis was then analyzed after 48-h transfection. Cell apoptosis assays were conducted in triplicate.

### Flow cytometric analysis

Transfected cells (5 × 10^5^) were fixed with 70 % ethanol and resuspended in 0.5 mL PBS, and then added with propidium iodide and 1 μg/mL RNase for 30 min. Processed samples were analyzed with a Beckman Coulter FC500 (Beckman Coulter, CA, USA). The experiments were performed in triple.

### Cell migration and invasion assays

For migration, transfected cells (1 × 10^4^) were plated into the upper chamber (BD Biosciences, San Jose, USA). For invasion, transfected cells (1 × 10^4^) were added to the upper chamber coated with matrigel (Millipore, Billerica, USA). RPMI-1640 containing 20 % FBS was plated into the lower chamber as a chemoattractant. After 24-h culture, membranes of the upper chamber were stained with 0.1 % crystal violet for 15 min. Migrated or invaded cells on the lower membrane were calculated under a light microscope (Olympus, Tokyo, Japan).

### Western blot analysis

Cellular protein lysates were isolated in a 10 % SDS-polyacrylamide gel and then transferred onto the polyvinylidene fluoride (PVDF) membranes (Bio-Rad, Hercules, USA). Membranes were blocked with 5 % non-fat dried milk containing antibody to p21 (Cell Signaling Technology, MA, USA, 1:1000), CyclinA1 (Abcam Biotechnology, USA, 1:1000) or GAPDH (Santa Cruz Biotechnology, CA, USA, 1:900) overnight at 4 °C. The membranes were then incubated with horseradish peroxidaselinked secondary antibody after washing with PBST. The proteins were visualized using ECL chemiluminescence. Bands were analyzed with Image J (National Institutes of Health, MD, USA).

### Statistical analysis

All experiments were independently repeated in triple. Data were expressed as mean ± standard deviation (SD) or frequencies and percentages if necessary. The *χ*^2^ test and Mann–Whitney *U*-test were used to investigate differences among groups of patients with low or high SNHG20 expression levels. The data from in vitro functional assays were analyzed by One-way ANOVA or Dunnett’s post-hoc test for multiple comparisons. Predictive value of SNHG20 was evaluated by the receiver operating characteristic curve (ROC) analysis. Kaplan-Meier method and log-rank test were used to assess the probability of overall survival (OS). Survival data were further estimated using the univariate and multivariate Cox proportional hazards model. Significant variables in univariate analyses were used in multivariate analyses according to the Cox regression analyses. *P* < 0.05 was chosen for statistical significance.

## Results

### LncRNA SNHG20 is up-regulated in human CRC tissues and cell lines

**Fig. 1:**
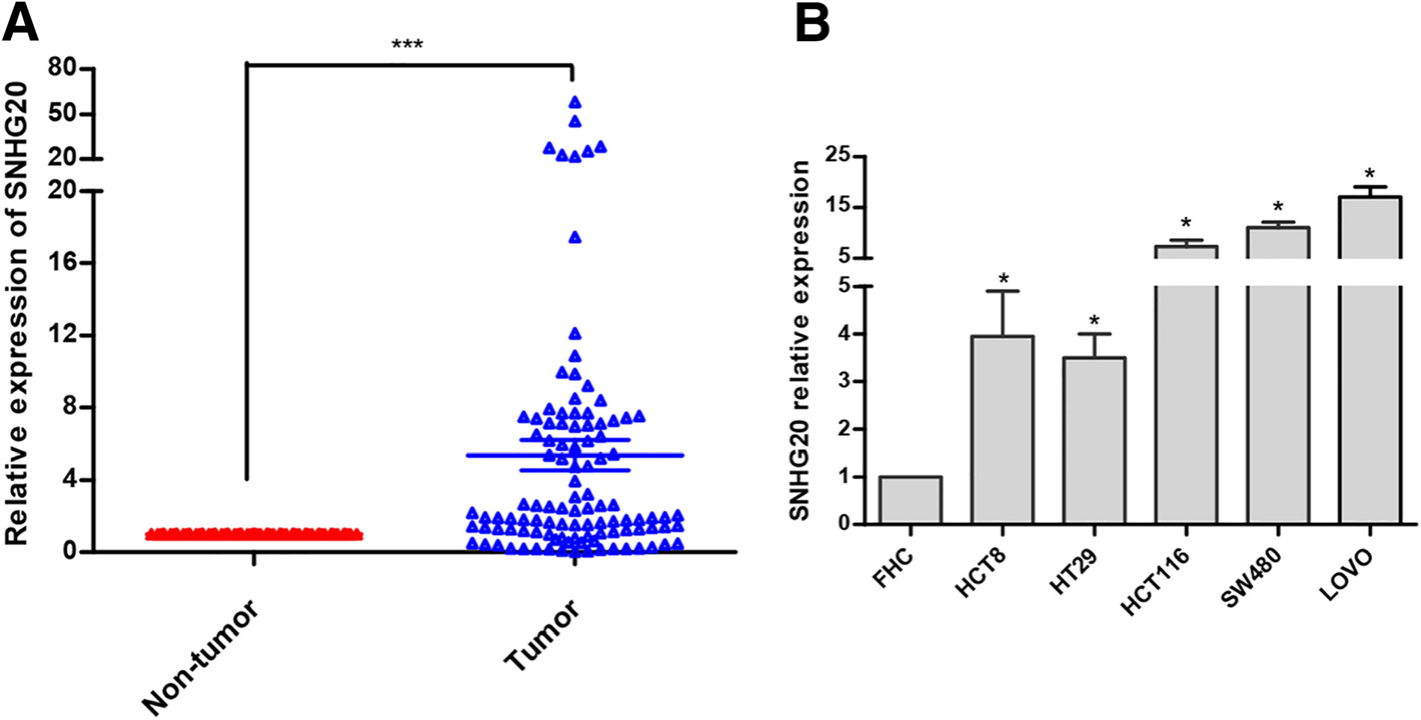
Relative SNHG20 expression in human CRC tissues and cell lines. **a** The expression of SNHG20 was measured by qRT-PCR in tumor and non-tumor tissues from 107 paired CRC samples. SNHG20 expression levels were normalized to GAPDH. **b** Relative expression of SNHG20 between five CRC cell lines (HCT8, HT29, HCT116, SW480, LOVO) and a normal intestinal epithelial cell line FHC. Each cell line was duplication analyzed three times. *P < 0.05, ***P < 0.001

To know the expression manner of SNHG20, we measured the expression of SNHG20 in 107 pairs of CRC and corresponding normal tissues by qRT-PCR. The results indicated that SNHG20 expression in tumor tissues was markedly higher than that in adjacent non-tumor tissues (*P* < 0.001, Fig. 1a). To further confirm its expression levels in CRC, we measured the levels of SNHG20 expression in FHC and CRC cell lines (HCT8, HT29, HCT116, SW480, and LOVO). We observed that CRC cell lines exhibited higher levels of SNHG20 compared with FHC cells (*P* < 0.05, Fig. 1b).

### Association between SNHG20 and clinicopathological features of CRC

**Fig. 2:**
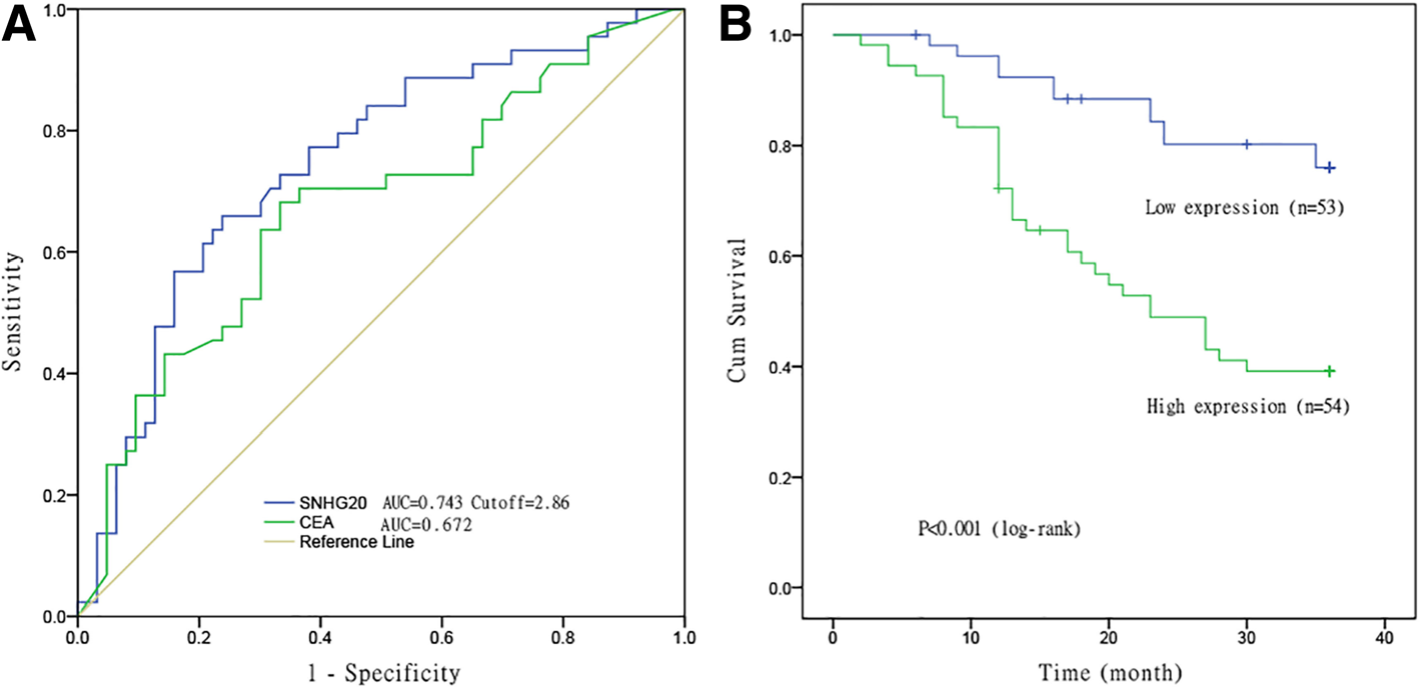
ROC curve analysis and prognostic significance of SNHG20 in CRC patients. **a** Receiver operating characteristic (ROC) curve analysis was used to determine whether SNHG20 is really a good candidate to discriminate tumor tissues from non-tumor tissues. **b** Kaplan–Meier survival curve analysis shows that patients with higher expression of SNHG20 had a shorter overall survival compared with those with lower expression of SNHG20. *P* value was assessed by log-rank test

To further understand the clinical significance of SNHG20 up-regulation in CRC patients, we carried out to identify potential correlations between SNHG20 expression and clinical characteristics of CRC. The main characteristics of 107 CRC patients are summarized in Table 1. Furthermore, the optimal cutoff value of SNHG20 expression was 2.86-fold for OS with the largest Youden’s index according to the relative expression levels of SNHG20 (Fig. 2a). All CRC patients were subsequently divided into two groups (high expression group ≥ 2.86 and low expression group < 2.86). The detailed relationships between SNHG20 expression manner and clinicopathological features of CRC patients are shown in Table 1. Interestingly, SNHG20 overexpression in CRC patients had a significant association with advanced TNM stage (*P* = 0.043), depth of invasion (*P* = 0.020), distant metastasis (*P* = 0.007), and CEA (*P* = 0.012).

### High expression of SNHG20 is correlated with poor prognosis of patients with CRC

Subsequently, survival analyses were conducted to assess the association between SNHG20 expression and overall survival of CRC patients by Kaplan-Meier survival curves and log-rank test. The results showed that the expression levels of SNHG20 were reversely associated with OS (*P* < 0.001, Fig. 2b). Furthermore, Cox regression analyses were conducted to evaluate the prognostic factors in 107 CRC patients. Univariate analysis showed that patients with depth of invasion (*P* = 0.040), distant metastasis (*P* < 0.001), tumor differentiation (*P* = 0.039), and high SNHG20 (*P* < 0.001) expression had markedly shorter overall survival (Table 2). Multivariate analysis further indicated that SNHG20 expression was a significant independent prognostic factor for CRC patients (HR = 2.97, 95 % CI = 1.51–5.82, *P* = 0.002). Additionally, SNHG20 expression also served as an independent indicator for non-metastatic patients with CRC in multivariate analysis (HR = 1.63, 95%CI = 1.22-3.98, *P* = 0.011, Table 3).

**Table 2:** Summary of overall survival analyses by univariate and multivariate COX regression analyses

Risk factors	Univariate analysis		Multivariate analysis	
	HR (95 % CI)	*P*	HR (95 % CI)	*P*
Sex	1.17 (0.61–2.24)	0.637		
Age (years)	0.92 (0.51–1.66)	0.774		
Tumor location	0.95 (0.51–1.77)	0.872		
T	8.01 (1.10–58.19)	**0.040**	4.04 (0.54–30.24)	0.174
N	1.62 (0.89–2.93)	0.114		
M	3.51 (1.93–6.37)	**<0.001**	2.83 (1.54–5.20)	**0.001**
Grade	2.02 (1.04–3.92)	**0.039**	0.99 (0.46–2.15)	0.981
CEA	1.75 (0.82–3.77)	0.152		
SNHG20 (high/low)	3.52 (1.81–6.85)	**<0.001**	2.97 (1.51–5.82)	**0cp002**

Statistical significance is highlighted by bold font

*T* depth of tumor, *N* lymph node, *M* distant metastasis, *CEA* carcino-embryonic antigen, *HR* hazard ratio, CI confidence, interval

**Table 3:** Summary of overall survival analyses by univariate and multivariate COX regression analyses in non-metastatic patients

Risk factors	Univariate analysis		Multivariate analysis	
	HR (95 % CI)	*P*	HR (95 % CI)	*P*
Sex	1.52 (0.56–4.09)	0.409		
Age (years)	0.34 (0.13–0.86)	**0.023**	0.42 (0.15–1.23)	0.113
Tumor location	1.50 (0.66–3.41)	0.330		
T	5.54 (1.05–41.11)	**0.034**	5.45 (0.68–43.51)	0.109
N	1.79 (0.79–4.08)	0.162		
Grade	1.44 (0.34–6.16)	0.623		
CEA	1.08 (0.45–2.64)	0.861		
SNHG20 (high/low)	2.32 (1.11–5.36)	**0.019**	1.63 (1.22–3.98)	**0.011**

Statistical significance is highlighted by bold font

*T* depth of tumor, *N* lymph node, *CEA* carcino-embryonic antigen, *HR* hazard ratio, CI confidence, interval

### Manipulation of SNHG20 expression levels in CRC cells

**Fig. 3:**
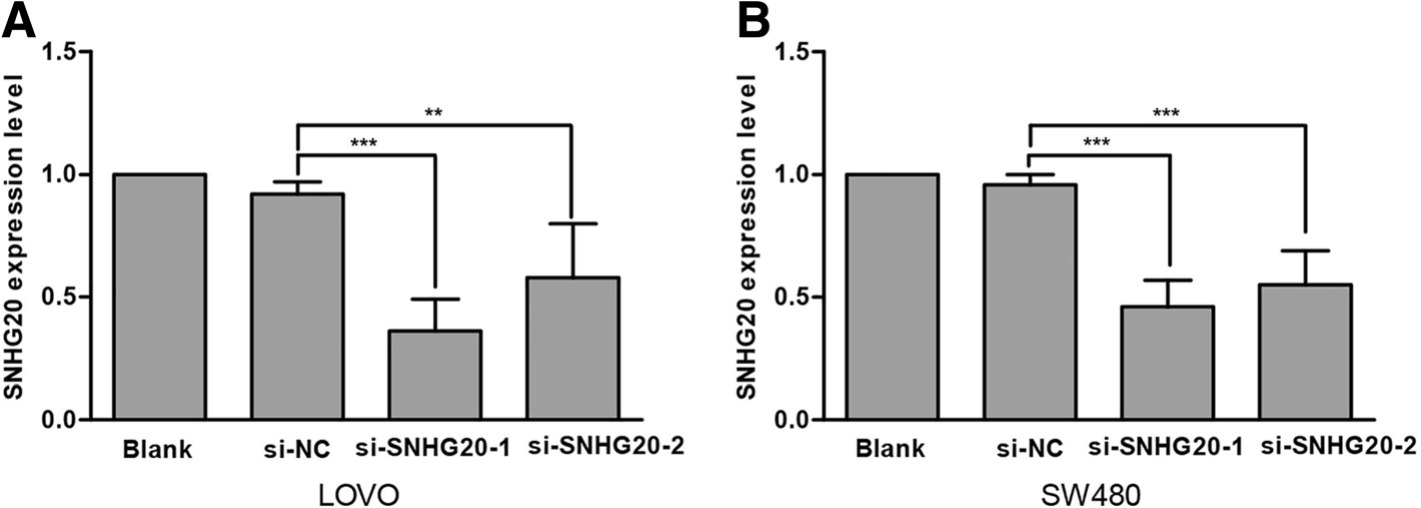
Manipulation of SNHG20 in CRC cells. QRT-PCR analyses of SNHG20 expression levels after transfection in LOVO (**a**) and SW480 (**b**) cells with si-SNHG20 or si-NC (negative control). **P < 0.01, ***P < 0.001

To assess the biological roles of SNHG20 in CRC, we examined the levels of SNHG20 expression in a variety of cells lines, and found that there were higher expression levels of SNHG20 in LOVO and SW480 cells. Therefore, we suppressed the endogenous expression of SNHG20 in LOVO and SW480 cells by siRNA to further explore the biological effects of SNHG20 on CRC cells. Two specific siRNAs of SNHG20 were synthesized and transfected into LOVO and SW480 cells. As shown in Fig. 3, si-SNHG20-1 effectively inhibited the expression of SNHG20 (*P* < 0.001). So, si-SNHG20-1 was selected for further study.

### Knockdown of SNHG20 affects biological behaviors of CRC cells

**Fig. 4:**
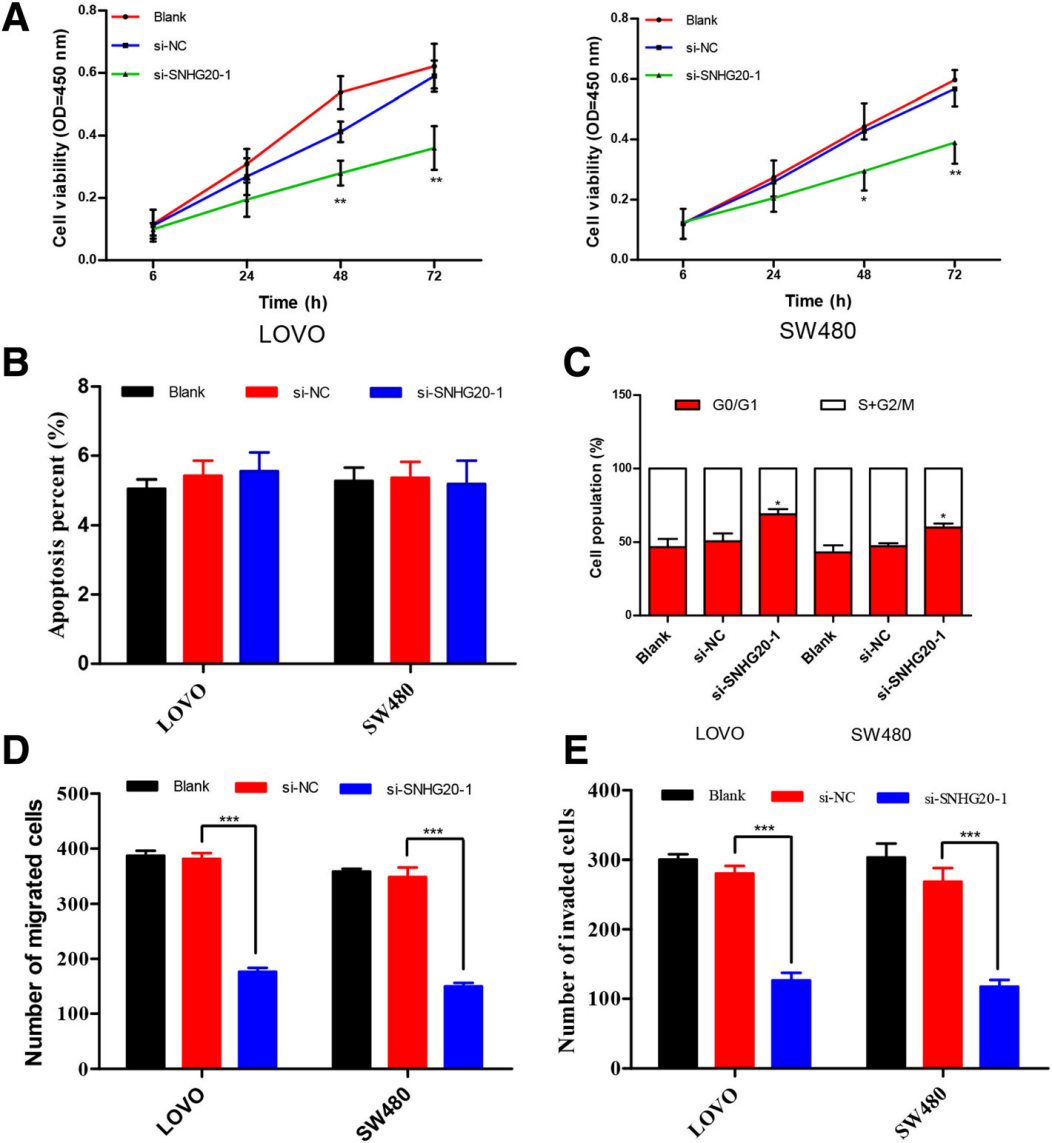
Influence of SNHG20 knockdown on CRC cells. **a** At 48 h after transfection, CCK8 assay was performed to determine the proliferation of LOVO and SW480 cells. **b** Cell apoptosis was analyzed via flow cytometry after 48-h transfection. **c** Cell cycle analysis of CRC cells transfected with si-NC or si-SNHG20. **d**-**e** Transwell assays were employed to assess the changes in migratory and invasive capabilities of CRC cells transfected with si-NC or si-SNHG20. The data are expressed as the mean ± SD. The assays are performed in triple. *P < 0.05, **P < 0.01, ***P < 0.001

To explore whether endogenous knockdown of SNHG20 inhibited proliferative capacity in CRC cells, CCK8 assay was performed. Growth curves determined by CCK8 assays revealed that cell proliferation was dramatically decreased by inhibition of SNHG20 expression in LOVO (Fig. 4a) and SW480 cells (Fig. 4b). To further probe potential mechanisms by which knockdown of SNHG20 attenuated CRC cell proliferation, we estimated cell cycle in CRC cell lines after SNHG20 knockdown by flow cytometric cell cycle assay. The results showed that SNHG20 knockdown led to a remarkable accumulation of CRC cells at G0/G1 phase and a significant reduction of cells at S + G2/M phase (*P* < 0.05, Fig. 4c). However, the proportion of apoptotic cells showed no significant difference among these groups (*P* > 0.05, Fig. 4b). Collectively, SNHG20-induced acceleration of CRC cells proliferation appeared to be mediated through modulation of cell cycle arrest, rather than apoptosis.

We also observed the impact of SNHG20 knockdown on CRC cells migration or invasion by Transwell assay. Our results showed that the SNHG20 knockdown suppressed cell migration by 53.7 % in LOVO cells, and by 55.1 % in SW480, respectively (*P* < 0.001, Fig. 4d). Matrigel invasion assays also illustrated that silence of SNHG20 in CRC cells caused a significant decrease in cell invasion (*P* < 0.001, Fig. 4e). These results revealed that SNHG20 affected cell migration and invasion in vitro.

### SNHG20 exerts its effect through cell cycle-associated proteins for promotion of cell cycle progression

**Fig. 5:**
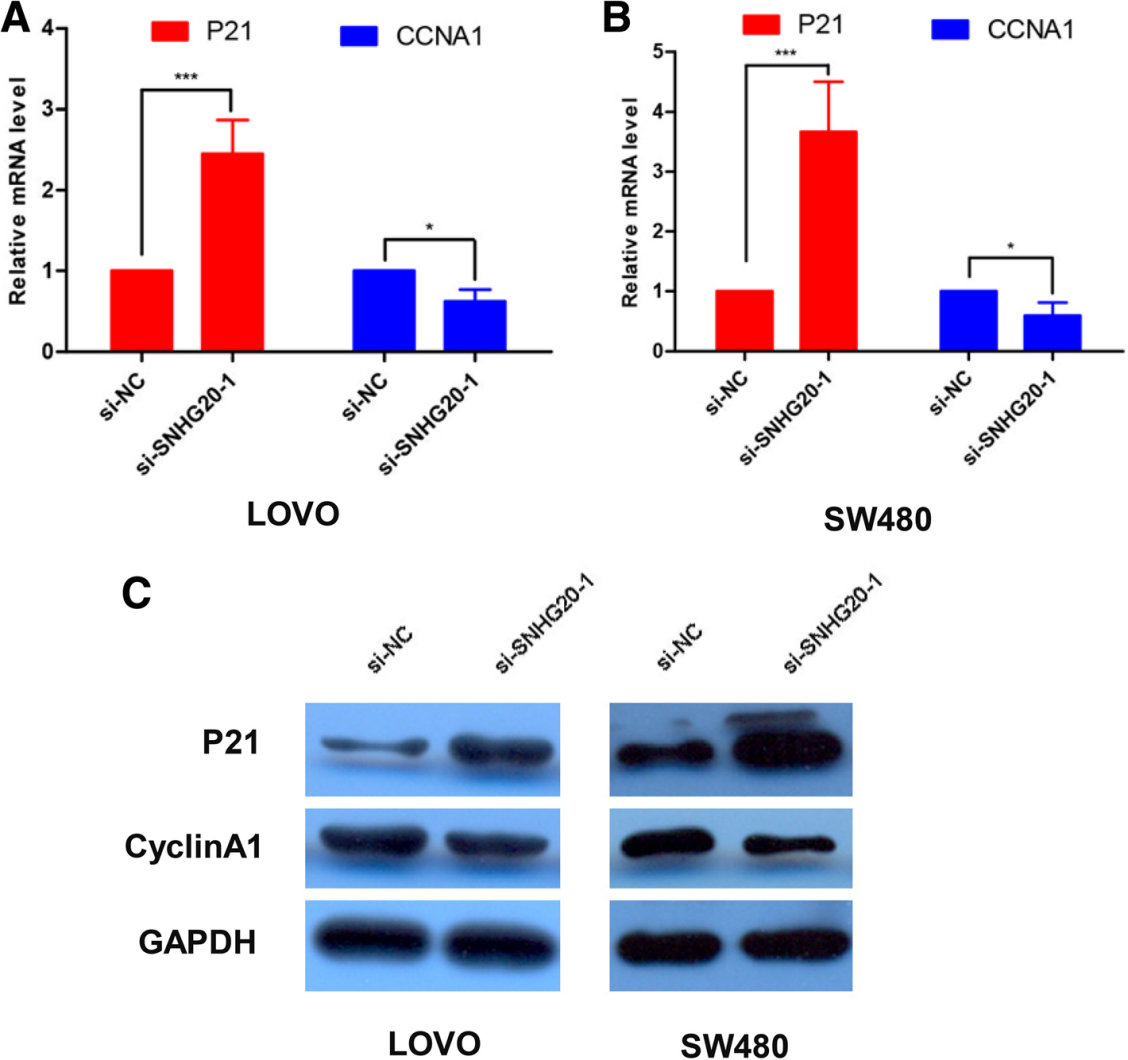
SNHG20 facilitates CRC cell growth by regulating cell cycle-associated genes and accelerating cell cycle progression. **a**-**b** mRNA expression levels of cell cycle-associated genes in CRC cells transfected with si-SNHG20 or si-NC cells for 48 h. **c** Protein expression levels of cell cycle-associated genes in CRC cells transfected with si-SNHG20 or si-NC cells for 48 h. The data are presented as the mean ± SD. All results are representative of three independent experiments. *P < 0.05, ***P < 0.001

As SNHG20 affected cells proliferation through modulation of the G1-S checkpoint, we examined cell cycle-regulatory gene expressions at the transcriptional and translational levels. The results showed that p21 mRNA and protein levels were significantly increased in CRC cells that were transfected with si-SNHG20-1 compared to those transfected with si-NC (Fig. 5a -c). Furthermore, si-SNHG20-1 attenuated CyclinA1 expression in both LOVO and SW480 cells (Fig. 5a - c). Taken together, our data suggest that SNHG20 promotes cell proliferation via the acceleration of the cell cycle progression.

## Discussion

Up to now, this is the first study about the roles of SNHG20 in human colorectal cancer. In the present study, we explored its clinical performance in patients with CRC. We observed that SNHG20 expression was upregulated in CRC tissues compared to corresponding normal tissues from 107 CRC patients. Our results showed that SNHG20 expression was correlated with TNM stage and serum CEA. In addition, high levels of SNHG20 expression were associated with worse OS and could be an independent prognostic indicator for CRC patients. Interestingly, knockdown of SNHG20 significantly suppressed cell proliferation, migration, invasion and cell cycle progression *in vitro* in CRC cells. Collectively, SNHG20 may act as an oncogenic function to be involved in carcinogenesis and development of CRC.

It has been confirmed that ectopic expression of lncRNAs has the capability to impact cellular functions through executing as signals, decoys, guides, and scaffolds [19, 20]. Furthermore, abnormal lncRNA function abolishes primary cell biology by inducing epigenetic depressions of downstream target genes [11]. For example, lncRNA H19 regulated Vimentin, ZEB1, and ZEB2 expression by competing with endogenous RNA (miR-138 and miR-200a), which resulted in the epithelial to mesenchymal transition (EMT) progression [21]. FEZF1-AS1, a long noncoding RNA upregulated in CRC, accelerated malignant development through FEZF1 induction [12]. Furthermore, accumulating evidence identified that lncRNA can serve as diagnostic and prognostic markers for a variety of cancer types including CRC [22, 23]. 91H expression in CRC may serve as a useful predictor for overall survival in patients with CRC [10]. A previous study has reported that SNHG20 overexpression in HCC patients, served as an independent prognostic predictor for patients with HCC [18]. All these results suggest that lncRNAs may be involved in the carcinogenesis and progression of CRC, which encourage us to investigate that SNHG20 may facilitate CRC malignant progression and serve as a novel diagnostic or therapeutic target for CRC.

Herein, we have identified that SNHG20 levels were overexpressed in CRC and may be considered as a predictor for CRC patients, which were consistent with the previous findings in HCC [18]. To further explore its biological roles in CRC, we determined the effect of SNHG20 on CRC cell biology. CRC cell lines LOVO and SW480 with highest SNHG20 expression were chosen to explore the influence of SNHG20 on CRC cell phenotype. Inhibition of SNHG20 in LOVO and SW480 cells led to a significant suppression of proliferation, migration and invasion as well as cell cycle arrest. However, the impact of SNHG20 knockdown on the apoptosis of CRC cells was not observed. These results indicated that knockdown of SNHG20 inhibits cell growth through blocking cell cycle progression in CRC cells. Therefore, SNHG20 may represent a promising target for CRC therapy.

To understand the potential molecular mechanisms by which SNHG20 promotes proliferation, migration and invasion of CRC, we assessed its potential target proteins involved in cell cycle progression. Here, loss of SNHG20 in CRC cells resulted in a marked decrease in Cyclin A1 mRNA and protein expression levels, while SNHG20 expression was inversely correlated with p21 expression.

Cyclin A1 is a protein that is encoded by the CCNA1 gene belonging to the highly conserved cyclin family in humans [24], which is shown to bind to some important cell cycle regulators, such as transcription factor E2F1, Rb family proteins, and the Kip/Cip family of CDK-inhibitor proteins [25]. Mounting evidence showed that Cyclin A1 alters cell cycle progression to induce carcinogenesis [26, 27]. p21, encoded by the CDKN1A gene located on 6p21.2 in humans, is a cyclin-dependent kinase inhibitor that inhibits the complexes of CDK2 and CDK1 to mediate the p53-dependent cell cycle G1 phase arrest [28–30]. Our results indicated that SNHG20 contributes to the proliferation of CRC cells via regulating Cyclin A1 and p21 expression. However, the precise molecular regulating how SNHG20 controls Cyclin A1 and p21 expression remains unclear and requires further investigation.

Several limitations should be acknowledged in the present study. Firstly, all samples were only collected from two hospitals, and the sample size was limited. Further studies should be needed to validate the correlations of SNHG20 expression with CRC. Secondly, this study was a retrospective analysis, which resulted in another limitation. These limitations will claim for a larger, prospective, randomized and multicenter study in the future. Additionally, our results did not validate how SNHG20 regulated cell cycle-associated proteins. So, further studies are necessary to elucidate the precise mechanisms by which SNHG20 modulates its targets.

## Conclusions

Our present work has demonstrated that SNHG20 expression is significantly upregulated in CRC tissues, suggesting that SNHG20 may be an adverse prognostic marker for CRC patients and a higher risk for cancer development. The present study showed that SNHG20 may regulate the ability of cell proliferation, invasion and migration through modulation of Cyclin A1 and p21 expression. Further insights into the clinical and functional implications of SNHG20 and its regulated pathways may facilitate the identification of new diagnostic or predictive indicators and drug targets for colorectal cancer.

## Abbreviation

CRC: Colorectal cancer
EMT: Epithelial to mesenchymal transition
HULC: Hepatocellular carcinoma upregulated long noncoding RNA
lncRNA: Long noncoding RNA
MALAT1: Metastasis-associated lung adenocarcinoma transcript 1
OS: Overall survival
ROC: Receiver operating characteristic curve
SNHG20: Small nucleolar RNA host gene 20
